# Inferring the direction of rhythmic neural transmission via inter-regional phase-amplitude coupling (ir-PAC)

**DOI:** 10.1038/s41598-019-43272-w

**Published:** 2019-05-06

**Authors:** Bijurika Nandi, Peter Swiatek, Bernat Kocsis, Mingzhou Ding

**Affiliations:** 1 J. Crayton Pruitt Family Department of Biomedical Engineering, Herbert Wertheim College of Engineering, University of Florida, Gainesville, FL USA; 2 Department of Psychiatry at BIDMC, Harvard Medical School, Boston, MA USA

**Keywords:** Cognitive neuroscience, Neural circuits

## Abstract

Phase-amplitude coupling (PAC) estimates the statistical dependence between the phase of a low-frequency component and the amplitude of a high-frequency component of local field potentials (LFP). To date PAC has been mainly applied to one signal. In this work, we introduce a new application of PAC to two LFPs and suggest that it can be used to infer the direction and strength of rhythmic neural transmission between distinct brain networks. This hypothesis is based on the accumulating evidence that transmembrane currents related to action potentials contribute a broad-band component to LFP in the high-gamma band, and PAC calculated between the amplitude of high-gamma (>60 Hz) in one LFP and the phase of a low-frequency oscillation (e.g., theta) in another would therefore relate the output (spiking) of one area to the input (somatic/dendritic postsynaptic potentials) of the other. We tested the hypothesis on theta-band long range communications between hippocampus and prefrontal cortex (PFC) and theta-band short range communications between dentate gyrus (DG) and the Ammon’s horn (CA1) within the hippocampus. The ground truth was provided by the known anatomical connections predicting hippocampus → PFC and DG → CA1, i.e., theta transmission is unidirectional in both cases: from hippocampus to PFC and from DG to CA1 along the tri-synaptic pathway within hippocampus. We found that (1) hippocampal high-gamma amplitude was significantly coupled to PFC theta phase, but not vice versa; (2) similarly, DG high-gamma amplitude was significantly coupled to CA1 theta phase, but not vice versa, and (3) the DG high-gamma-CA1 theta PAC was significantly correlated with DG → CA1 Granger causality, a well-established analytical measure of directional neural transmission. These results support the hypothesis that inter-regional PAC (ir-PAC) can be used to relate the output of a rhythmic “driver” network (i.e., high gamma) to the input of a rhythmic “receiver” network (i.e., theta) and thereby establish the direction and strength of rhythmic neural transmission.

## Introduction

Neural information processing depends on interactions between ensembles of neurons. Being able to assess the patterns of neuronal interactions is thus essential for a better understanding of the cooperative nature of neuronal computation. Neural interactions are directional. Whereas cross correlation function in time domain and coherence function in spectral domain provide useful information regarding functional connectivity, they are technically symmetric in that when signal A is coherent with signal B, signal B is equally coherent with signal A, thereby failing to provide information on the direction of neural transmission. Cross-frequency coupling (CFC) is a measure increasingly used to quantify the relation between neuronal activities in different frequency bands within a single local field potential (LFP). When applied to pairs of LFP signals recorded in distinct structures^1^, CFC is inherently asymmetric; it can associate one feature in one LFP with a different feature in the other LFP. Based on this property of CFC, we advance here a new interpretation of this quantity, and suggest that it can be used to infer the direction and strength of rhythmic neural transmission between distinct oscillatory brain networks.

There are several CFC measures, including phase-phase coupling^2,3^, amplitude-amplitude coupling^4,5^, and phase-amplitude coupling. Among these measures, phase-amplitude coupling (PAC) estimates the statistical dependence between the phase of a low frequency oscillation and the amplitude of a high frequency oscillation, and is the most commonly applied CFC measure in neuroscience^6–14^. When applied to a single LFP recording, PAC results are often interpreted as a measure of how low-frequency fluctuations in membrane potential influence higher-frequency oscillations, namely, greater activity of excitatory and inhibitory neuron populations generates stronger postsynaptic potentials at high frequencies. We note that the implicit assumption here that PAC represents the influence of low-frequency phase on high-frequency amplitude does not necessarily apply in all situations; the opposite influence may take place in a two-LFP scenario in which high-frequency activity of one LFP, taken to reflect population spiking, can cause low-frequency oscillations in the membrane potential of the other LFP. In particular, when the pair of LFP signals are recorded in distant structures where establishing inter-regional synchrony requires the transmission of spike activity, the second interpretation is likely more accurate.

Owing to the ambiguity of the origin of LFP^15^, the interpretation of PAC is not always single-faceted. LFP is mainly generated by somatic/dendritic postsynaptic potentials, i.e., coordinated transmembrane currents summed across neurons; as such, it may also contain wide-band signals resulting from spikes and spike afterpotentials^16,17^. Neuronal spikes were shown to correlate with gamma-band power in cognitive tasks^18,19^. There is increasing evidence indicating that LFP contains broad-band high-gamma (>60 Hz) components generated by fast transient transmembrane currents related to action potentials^20,21^. Buzsaki and Wang (2012) even noted that, “although spike contamination can be a nuisance, by using proper analytical methods, spike power can be exploited as a proxy for the assessment of neuronal outputs even in recordings of LFPs”.

**Figure 1:**
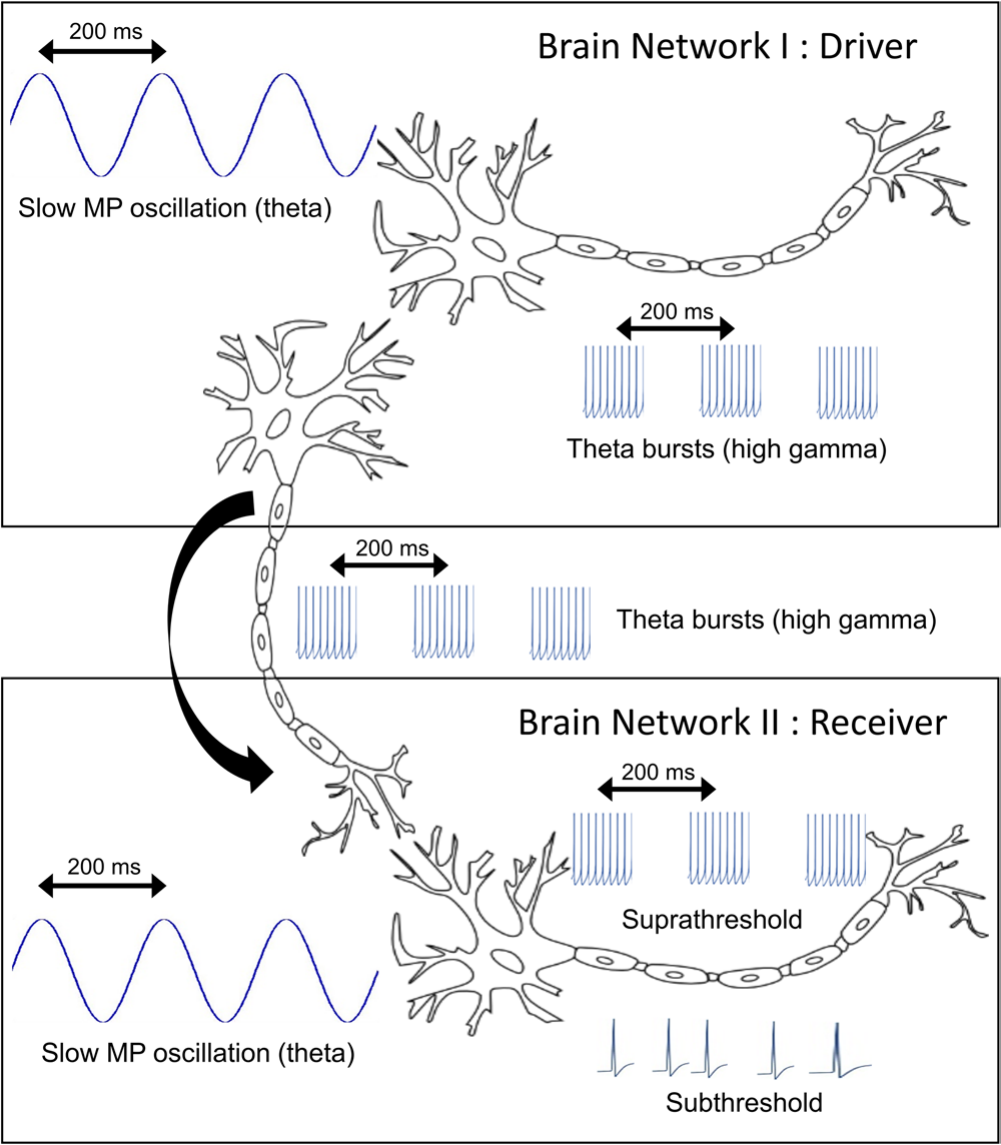
A pair of brain networks forming a driver-receiver relationship. In Brain Network I, slow rhythmic (e.g., theta) fluctuations of the membrane potential (MP) of principal neurons are suprathreshold and give rise to the theta rhythmic firing of action potentials (theta bursts), generating the low and high-frequency components of LFP recorded within the driver network, respectively, and leading to significant local PAC (l-PAC). When the theta-rhythmic spike trains are transmitted from Brain Network I to Brain Network II, the somatic/dendritic MP responses to the theta-rhythmic input are theta rhythmic as well, and give rise to the theta component of LFP recorded within this receiver network, leading to significant ir-PAC. In Brain Network II, depending on whether the MP fluctuations are suprathreshold or subthreshold, the l-PAC may or may not be significant.

The foregoing laid the foundation for the proposed extension of PAC analysis from a single LFP to two LFPs, in which we use the fast component in the high-gamma range as an indicator of spike activity representing the output of one network and test whether it correlates with low-frequency oscillations representing the input of the downstream targets of this network. In particular, we hypothesized that for two neural networks with a driver-receiver relationship, PAC calculated from two LFP recordings, referred to inter-regional PAC (ir-PAC) here, will be significant in one direction only, i.e., between high-gamma amplitude of the “driver” network, representing rhythmically modulated spike trains, and the phase of the low-frequency oscillation in the “receiver” network, representing the somatic/dendritic postsynaptic potentials in response to the driving spike train input (see Fig. 1 for a schematic illustration). We tested this hypothesis on different datasets involving simultaneous LFP recordings from PFC and different regions of the hippocampus during theta rhythm where directionality is well-established by abundant anatomical and physiological evidence. We predicted that hippocampal high-gamma amplitude was significantly coupled to theta phase in prefrontal cortex (PFC), but not vice versa, and high-gamma amplitude in dentate gyrus (DG) was significantly coupled to Ammon’s horn (CA1) theta phase, but not vice versa, in agreement with known anatomical connections predicting unidirectional theta transmission from hippocampus to PFC^22–24^ and from DG to CA1 along the tri-synaptic pathway within the hippocampus, but not back. Here, along the tri-synaptic pathway, granule cells of DG project to CA3 via mossy fibers, and pyramidal cells of CA3 provide major input to CA1 through Schaffer collaterals, namely, DG → CA3 → CA1^25,26^. For data where the appropriate experimental conditions were met^27^, the direction of DG → CA1 theta drive was also verified by Granger causality (GC)^28^, a well-established analytical measure of directional neural transmission^27^. For further support, local PAC, denoted l-PAC, was also calculated between theta frequency and high-gamma amplitude in each of the two recorded structures. We predicted that l-PAC was significant in the “driver” circuit, indicating that rhythmic membrane potential oscillations drive rhythmically synchronized spike train output, but significant l-PAC may or may not appear in the “receiver” circuit, depending on whether the rhythmic postsynaptic potentials was subthreshold or suprathreshold.

## Results

Our theoretical consideration behind ir-PAC and l-PAC is illustrated in Fig. 1 for a pair of brain networks forming a driver-receiver relationship. In Brain Network I (driver network), action potentials bursting at the theta frequency contribute to the high gamma component of the LFP recordings in the driver network. When these actions potentials are transmitted from Brain Network I to Brain Network II (receiver network), the membrane potential (MP) responses in Brain Network II to the action potential input contribute to the theta component within the LFP recordings in the receiver network. The proposed ir-PAC, assessing the coupling between high gamma amplitude in Brain Network I and theta phase in Brain Network II, will be statistically significant. In addition, in Brain Network I, l-PAC is also statistically significant, indicating that the rhythmic fluctuations of membrane potentials drive the theta rhythmic firing of action potentials. In Brain Network II, depending on whether the membrane oscillations are suprathreshold or subthreshold, the local PAC, denoted l-PAC, may or may not be statistically significant; in other words, the action potential firing patterns in the receiver network may or may not be theta rhythmic.

**Figure 2:**
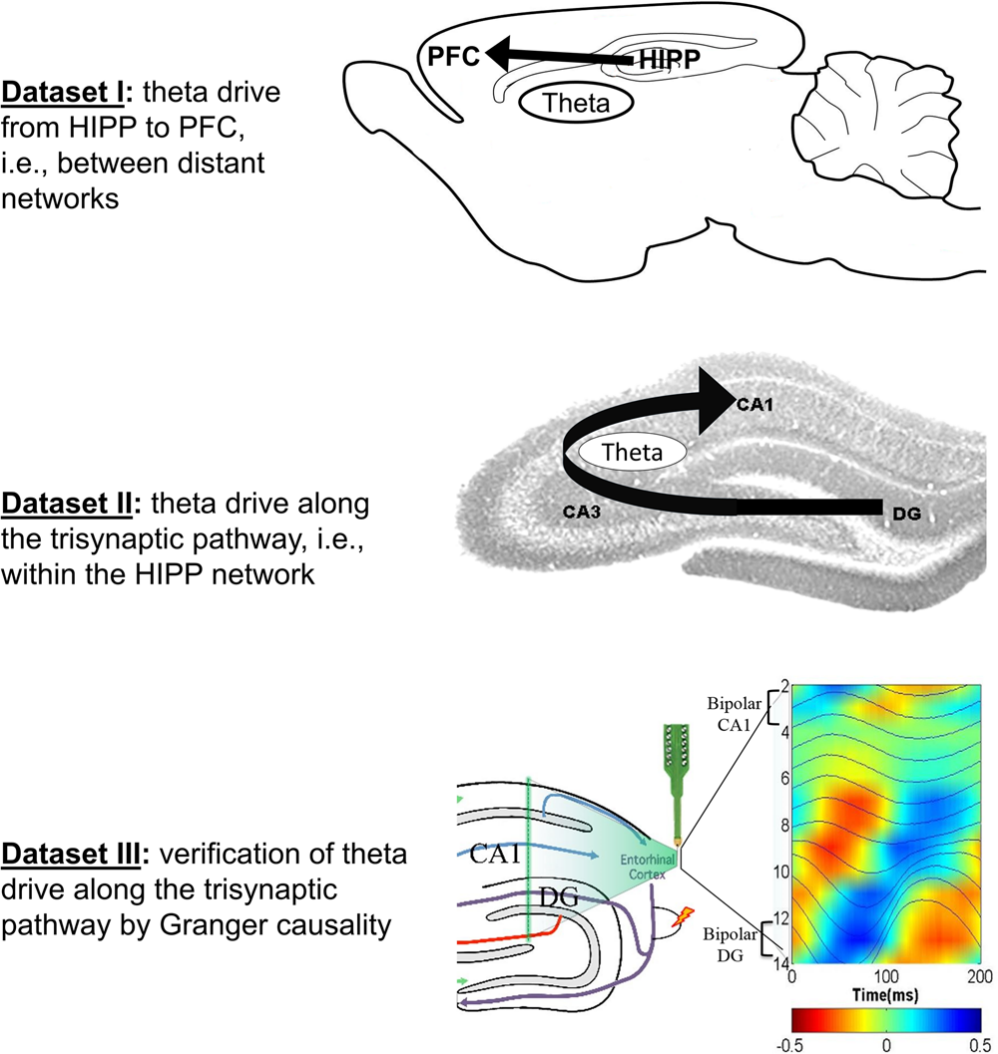
Schematics of analysis used in this study to test the hypothesis that ir-PAC across distinct brain networks can be used to estimate directionality of neural transmission between them. We used 3 datasets, with hippocampal theta drive spreading to the PFC (distant network) carried by rhythmic firing along the hippocampus (HIPP) → PFC pathway (Dataset I) or within the hippocampus along the classic trisynaptic pathway from the electrode placed below the fissure to the electrode above the CA1 pyramidal layer (Datasets II and III). Datasets I and II included unipolar LFP recordings in two locations during theta states (waking exploration and REM sleep) in freely moving rats. Dataset III compared ir-PAC and directions estimated from Granger causality (GC) using theta LFP evoked by brainstem stimulation and recorded by a 16-channel silicon probe across CA1 and DG in urethane-anesthetized rats. Theta dipoles in DG and CA1 were identified using perforant path stimulation, and PAC and GC was calculated between bipolar recordings, based on current source density analysis^27^. The background image in Fig. 2 (top) is adapted from “Distribution of relaxin-3 neurons and projections” by Craig Smith; permission is granted under CC BY. The background image in Fig. 2 (middle) is adapted from “Hippocampal Regions” by Semiconscious, which has been released into the public domain. Figure 2 (bottom) is adapted from Fig. 2 of ^27^; permission is granted under CC BY.

We tested the possible utility of using ir-PAC to infer direction of rhythmic neural transmission on data recorded from brain networks exhibiting theta rhythm. See Fig. 2 for illustrations of the three datasets. Theta, known to originate in the hippocampus and transmitted from the hippocampus to other parts of the brain in a behavior- and state-dependent manner^29,30^, has been intensively studied, and the fund of knowledge in the extant literature provides the basis for evaluating the performance of the proposed method.

Amplitude-phase pairing in PAC calculation, i.e., high gamma amplitude in the driver network with theta phase in the receiver network or vice versa, was calculated to examine whether it corresponded to known anatomical connections, and whether it matched the direction and strength identified by Granger causality when the dataset was appropriate for a GC analysis. In the first two datasets, we used unipolar LFP recordings in hippocampus and PFC (Dataset I) and in DG and CA1 (Dataset II) as examples of theta coupling between distant structures and between local networks within the hippocampus, respectively. These two datasets were recorded in freely moving rats during behavioral theta states of awake exploration and REM sleep. In the third dataset (Dataset III), we used 16-channel laminar LFP recordings across hippocampal layers, extending from above the pyramidal layer of CA1to DG below the hippocampal fissure. In this recording setup, bipolar LFP derivations representing theta dipoles elicited by brainstem stimulation in urethane anesthetized rats were made to allow precise measurement of GC^27^, which was then used for verification of the direction and strength of theta drive within the hippocampus estimated by ir-PAC (Fig. 2).

**Figure 3:**
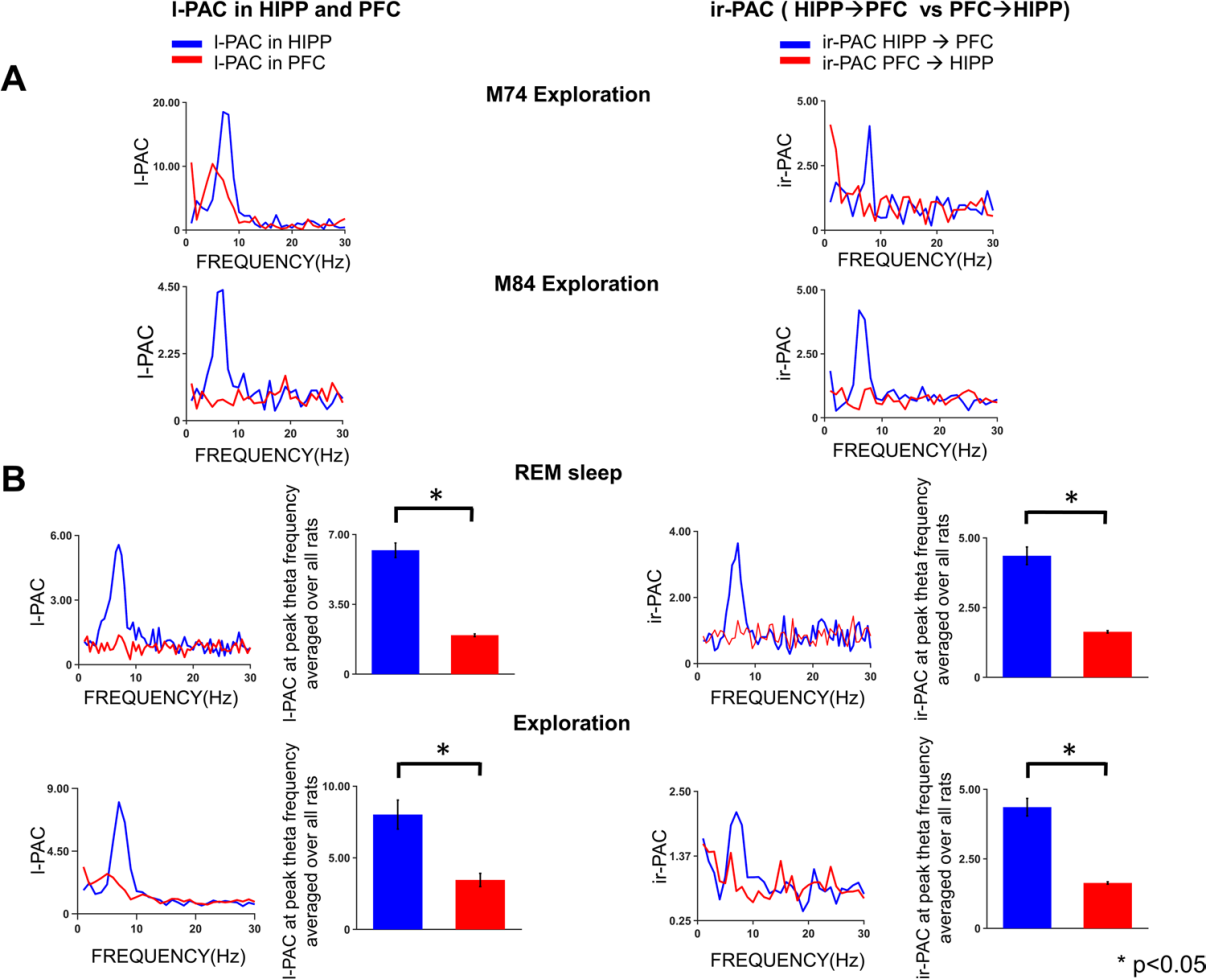
Theta-high gamma ir-PAC in two distant structures, hippocampus and PFC. *Left:* Local PAC (l-PAC) calculated using low frequency phase (0–30 Hz) and high gamma amplitude (65–90 Hz) of the same LFP signal in hippocampus (HIPP) and prefrontal cortex (PFC). *Right:* interregional PAC (ir-PAC) calculated using low frequency phase of one LFP signal and high gamma amplitude of the other LFP signal recorded in two different locations. (**A**) Two examples showing strong local theta-high gamma PAC in hippocampus and weaker (expt. M74) or no theta-high gamma PAC (expt. M84) in PFC during wake exploration. Note the strong hippocampal high gamma and PFC theta ir-PAC in both examples but weak PFC high gamma and hippocampal theta ir-PAC. (**B**) Average l-PAC (n = 6) and ir-PAC (n = 6) during REM sleep (*upper*) and exploration (*lower*). Note the strong l-PAC in hippocampus but weak theta PAC in PFC. In addition, the theta rhythmic transmission from hippocampus to PFC (HIPP → PFC) is captured by significant ir-PAC between hippocampal high gamma and PFC theta.

In Dataset I, analysis of LFP recordings in the hippocampus and PFC during natural theta states tested the possible theta drive between two distant structures along the anatomically demonstrated unidirectional hippocampo-prefrontal pathway^22–24^. There was strong local theta versus high-gamma PAC, l-PAC, in hippocampal recordings of all experiments, both during awake exploration and REM sleep (Fig. 3), corresponding to known synchronized theta fluctuations in membrane potential and in firing activity during these states^31,32^. In contrast to l-PAC in hippocampus, l-PAC in the PFC was low (Fig. 3B), where no significant coupling between PFC theta and PFC high gamma was found in the REM segments of 6 recordings, even though it was significant during waking occasionally in individual experiments (in 3 out of 6 recordings) (see, e.g., Experiment M74 in Fig. 3A), possibly depending on behavior which was not controlled in these experiments. The ir-PAC revealed significant coupling (p < 0.05) across hippocampal and PFC LFPs during theta states of both waking and REM sleep. Specifically, ir-PAC was high (significant coupling in 4 out of 6 recordings) between hippocampal high-gamma amplitude and PFC theta phase (ir-PAC = 3.2 ± 1.5 and 4.4 ± 1.9 in awake and REM, respectively) but was not significant when calculated using high-gamma amplitude in PFC and theta phase in hippocampal recording (ir-PAC = 1.6 ± 0.7 and 1.6 ± 0.2 in awake and REM, respectively), indicating that the hippocampal high gamma and PFC theta ir-PAC showed the significant hippocampus → PFC theta drive, as predicted; this finding was in agreement with the known anatomy and physiology of the theta rhythm, namely, it is primarily generated in the hippocampus, and propagated to PFC via the hippocampus to PFC connections.

**Figure 4:**
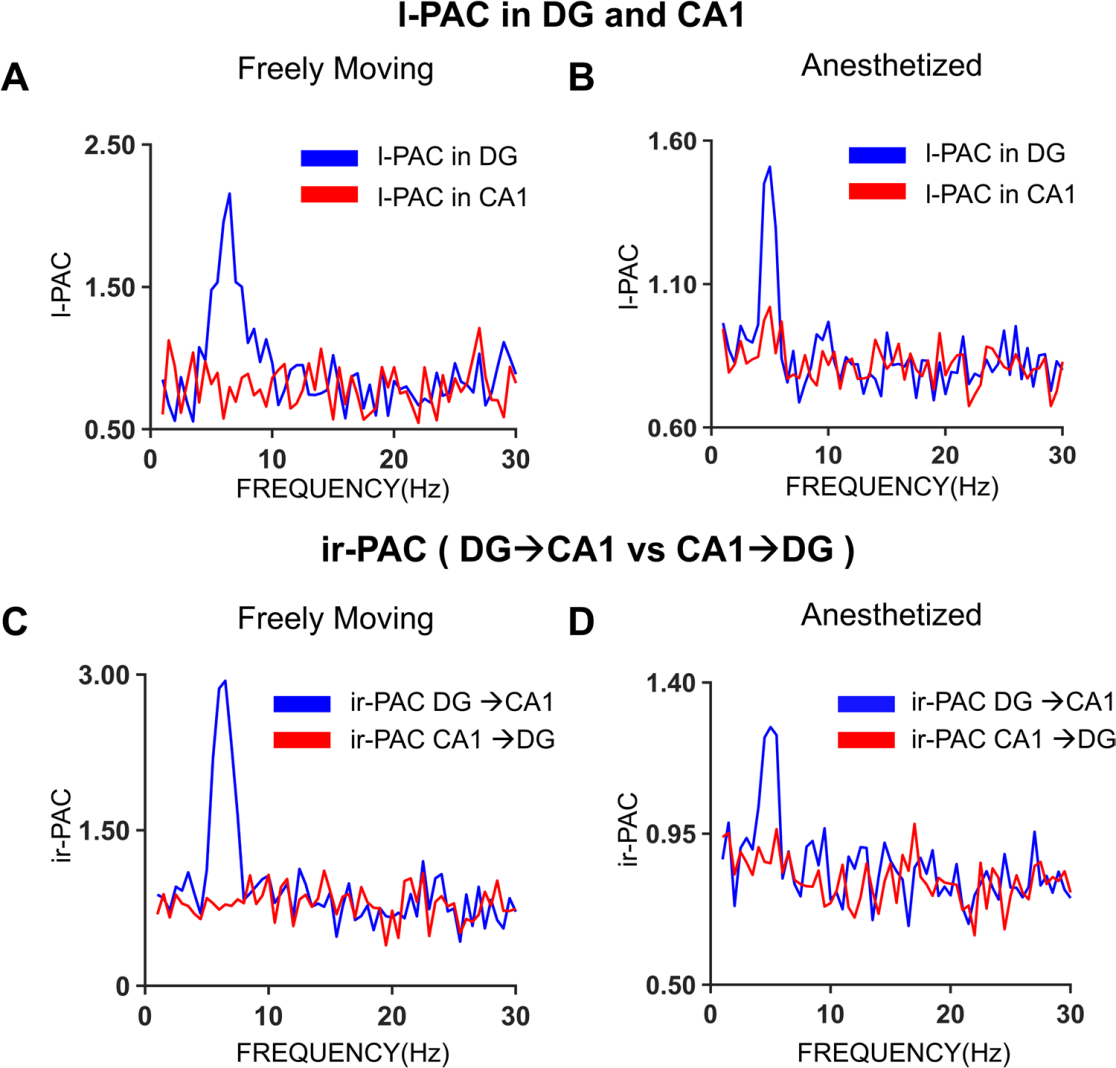
Theta-high gamma ir-PAC between hippocampal areas of DG and CA1. l-PAC between gamma amplitude (65–85 Hz) and low frequency phase in the frequency range of 1–30 Hz calculated using the same LFP signal in DG and CA1, averaged across (**A**) experiments (n = 13) with unipolar recordings of two LFP signals in freely moving rats in natural theta states of awake exploration and REM sleep and (**B**) experiments (n = 9) with bipolar recordings in the two major theta dipoles (derived from 16-channel LFP recordings across hippocampal layers of CA1 and DG) activated by brainstem stimulation under urethane anesthesia. ir-PAC between DG high gamma amplitude and CA1 low frequency phase and between CA1 high gamma and DG low frequency phase, averaged across (**C**) experiments (n = 13) in freely moving rats and (**D**) experiments (n = 9) in urethane-anesthetized rats.

Next, we analyzed ir-PAC across different subnetworks within the hippocampus in freely moving rats in the natural theta states of exploration and REM sleep (Dataset II) and in rats in which theta was elicited by brainstem stimulation under urethane anesthesia (Dataset III). In both datasets, the amplitude of high gamma in DG was significantly coupled to the phase of theta oscillations in CA1, but not vice versa, implying unidirectional theta transmission from DG to CA1 but no theta transmission in the opposite direction from CA1 to DG (Fig. 4). Thus, in both freely moving and anesthetized rats, significant ir-PAC (n = 9 for Dataset II and n = 2 in Dataset III) existed across areas between DG high-gamma amplitude and CA1 theta phase (ir-PAC = 3.6 ± 1.6 and 1.6 ± 0.8 in Datasets II and III, respectively), but not vice versa (ir-PAC = 1.5 ± 0.4 and 1.3 ± 0.4). The direction identified by ir-PAC corresponded to the transmission along the unidirectional intrahippocampal trisynaptic DG → CA3 → CA1 pathway, and the lack of anatomical projection from CA1 to DG^33–36^. It is noteworthy that the results were similar in the two datasets (Dataset II and III), even though PAC in Dataset II was calculated among unipolar hippocampal LFP recordings obtained with two electrodes placed in or above the CA1 pyramidal layer and below the hippocampal fissure in the DG respectively, whereas in Dataset III, LFPs in the two major theta dipoles were used utilizing bipolar recordings derived from 16-channel laminar LFPs. Also, in both Datasets II and III, the amplitude of gamma oscillations in DG was significantly coupled locally to the phase of theta oscillations of DG itself (l-PAC = 2.9 ± 1.5 and 1.8 ± 1 in Datasets II and III, respectively), whereas l-PAC was not significant in CA1 (l-PAC = 1.8 ± 0.5 and 1.3 ± 0.3 in Datasets II and III, respectively) (Fig. 4A).

**Figure 5:**
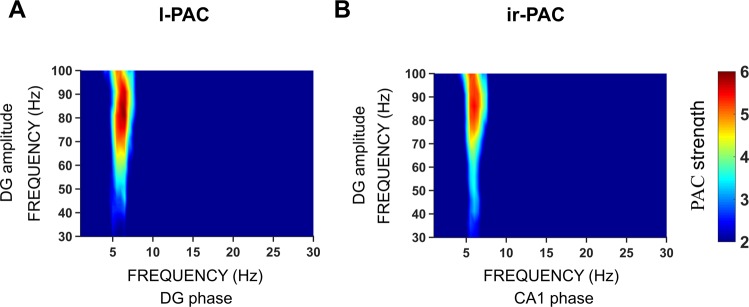
Role of wide-band gamma components in l-PAC and ir-PAC. Example of theta-gamma l-PAC in DG (**A**) and ir-PAC between DG and CA1 (DG → CA1) (**B**) in a freely moving rat. Comodulograms were calculated between gamma amplitude (30–100 Hz) and theta phase (1–30 Hz). Note that in both l-PAC and ir-PAC strong couplings are limited to narrow theta band (5–7 Hz) but are distributed over broad high gamma band (>60 Hz).

In the foregoing analysis, we specifically focused on the upper gamma band (>60 Hz) as a proxy of spiking activity^20,21^. Over the entire gamma range of 30–100 Hz, theta-gamma PAC was high only in the high-gamma band with peak in the 65–85 Hz range, considerably exceeding theta-gamma PAC in the low gamma range (<55 Hz) (Fig. 5). It is known, in contrast, that membrane potential oscillations generated by multiple mechanisms in the gamma range involving different types of interneurons and serving different memory functions appear in narrower gamma bands distributed however both around 40 Hz and at higher frequencies^8,15^.

**Figure 6:**
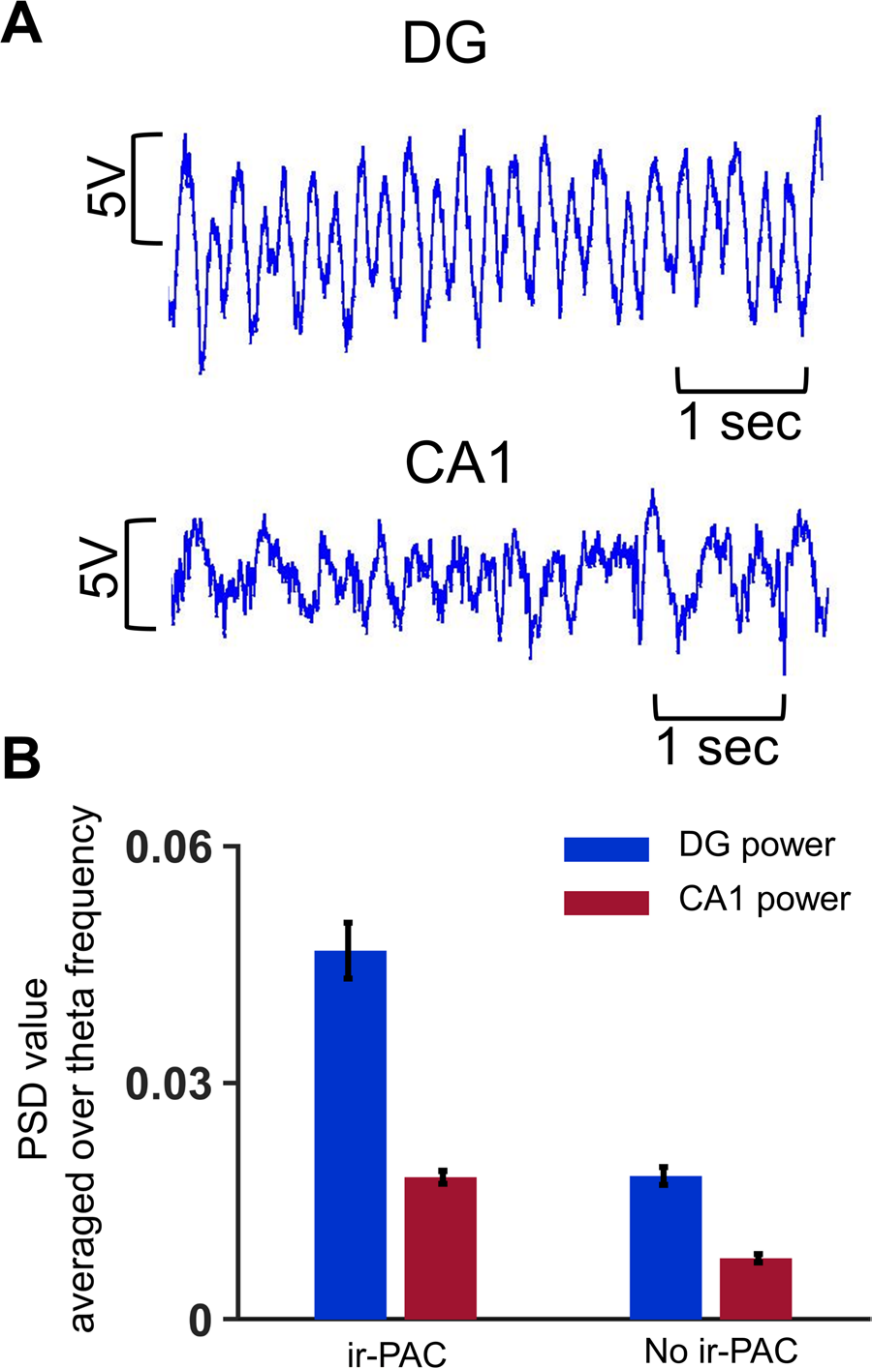
Relationship between theta power in DG and CA1 and theta-high gamma ir-PAC across DG and CA1. (**A**) Sample LFP traces (6 s) recorded in DG and CA1. (**B**) Average DG and CA1 power spectral density (PSD) in experiments with significant ir-PAC (n = 9) and not significant (n = 4) ir-PAC between DG high gamma amplitude and CA1 theta phase.

The existence of significant theta-high gamma PAC was also strongly influenced by the strength of theta oscillations. To show this, we divided individual LFP recordings in Dataset II into two groups: (1) which had significant ir-PAC between DG high gamma amplitude-CA1 theta phase (n = 9) and (2) which did not show any significant ir-PAC, either across areas or locally in the driver network DG (n = 4). Average theta power in recordings that showed significant ir-PAC had higher power in both DG and CA1 than in recordings that showed no significant ir-PAC (Fig. 6).

**Figure 7:**
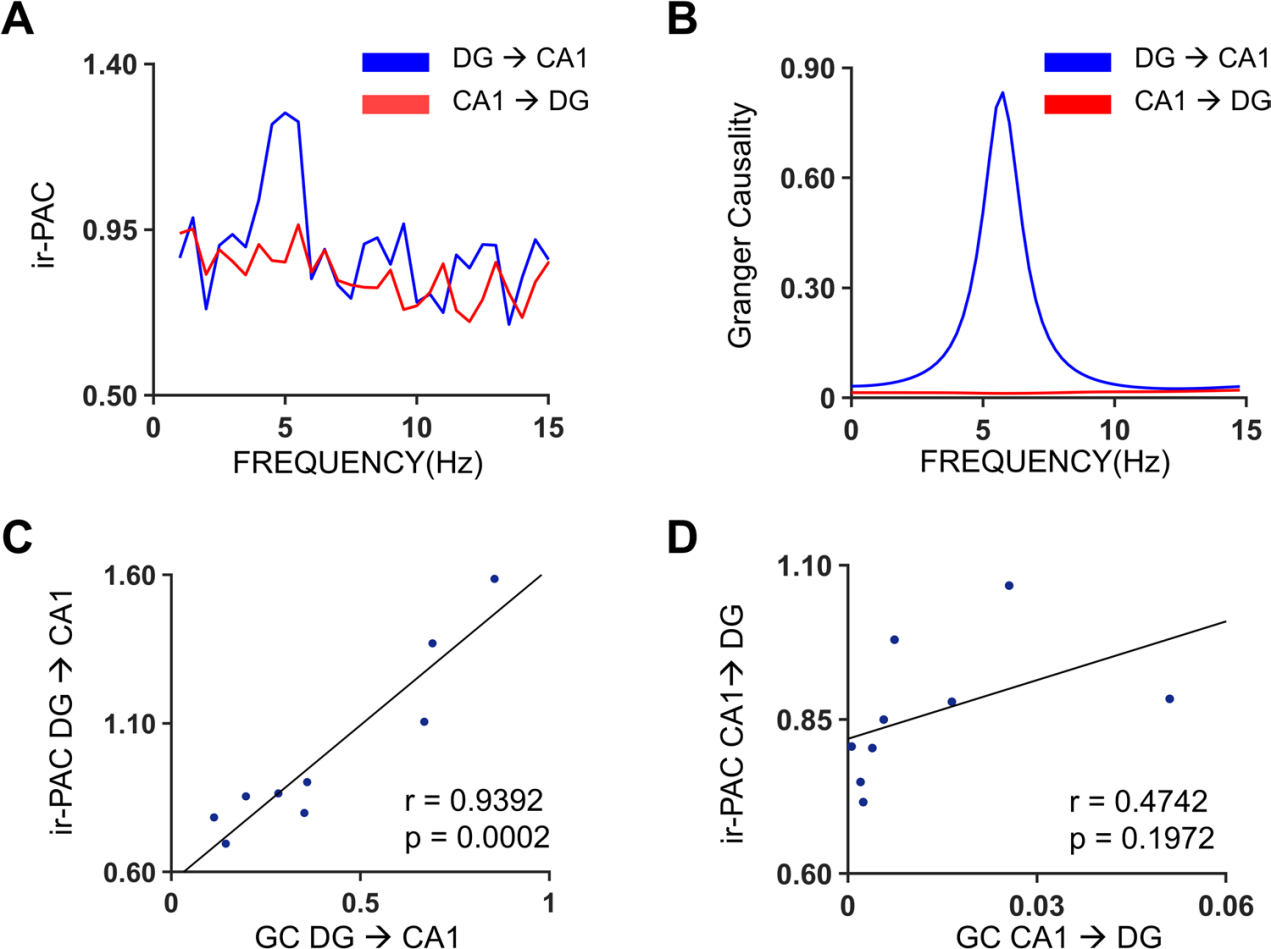
Comparison of directionality measures ir-PAC and Granger causality (GC). (**A**) Average ir-PAC strength between DG high gamma amplitude and CA1 low frequency phase in the frequency range of 1–30 Hz and vice versa (n = 9). (**B**) Average GC spectra between DG and CA1 (n = 9). (**C**) Correlation between theta-band GC (DG → CA1) and ir-PAC between DG high gamma amplitude and CA1 theta phase of the 9 recordings. (**D**) Correlation between GC (CA1 → DG) and ir-PAC between CA1 high gamma amplitude and DG theta phase of the 9 recordings.

**Figure 8:**
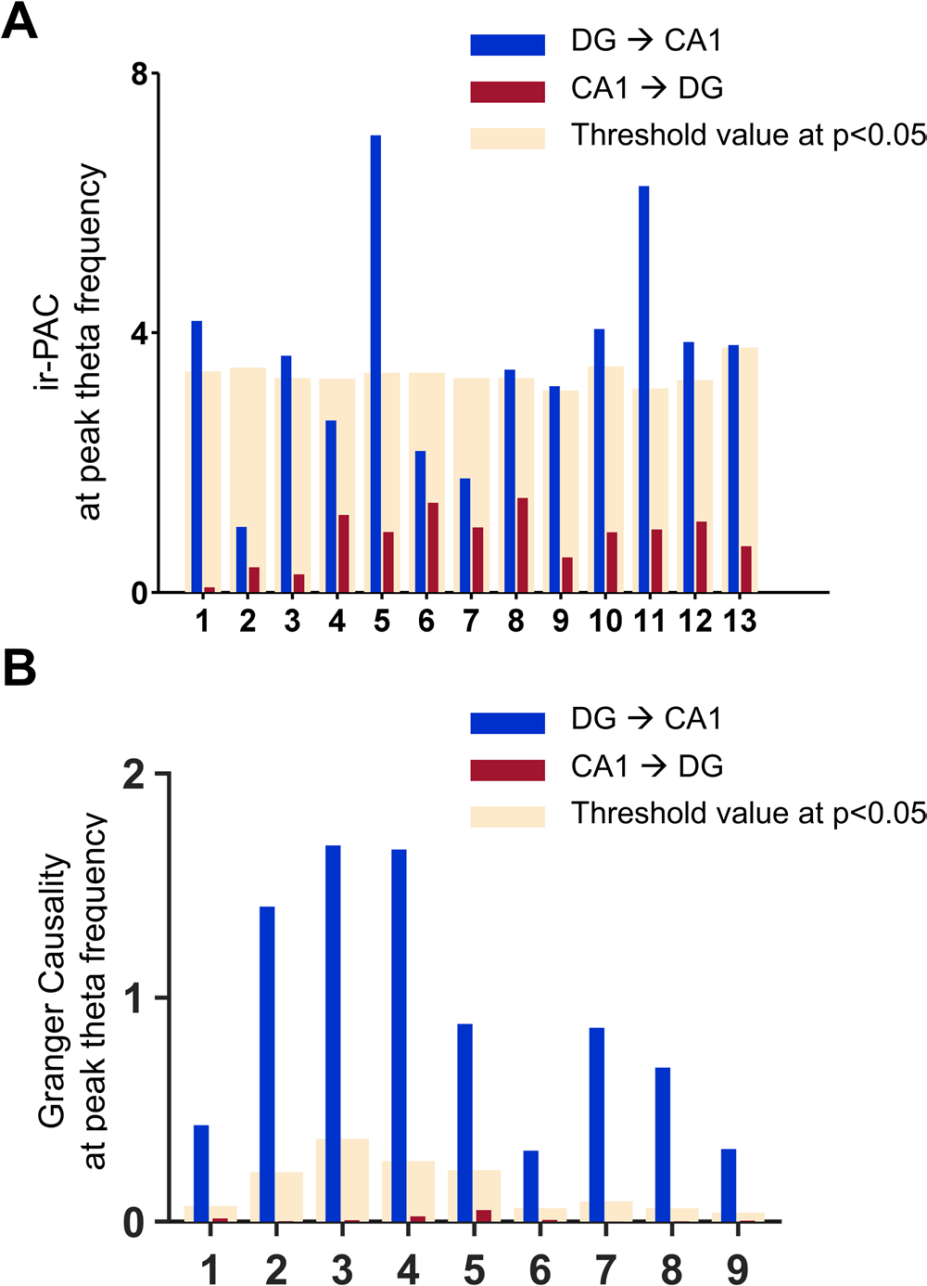
Comparison of estimates of the direction of within-hippocampal theta drive using ir-PAC of monopolar wire electrode recordings with GC calculated on bipolar recordings. (**A**) Bar plots showing ir-PAC between DG high-gamma amplitude and CA1 theta phase (blue) for 13 unipolar wire recordings (Dataset II) as well as ir-PAC between CA1 high-gamma amplitude and DG theta phase (red); significance threshold (p < 0.05) was indicated for each recording (cream). (**B**) Theta-band DG → CA1 GC (blue) and CA1 → DG GC (red) for the 9 bipolar recordings (Dataset III) recordings; significance threshold value at p < 0.05 (cream).

For Dataset III, owing to the electrode configuration, bipolar derivations can be made for the theta dipoles, which enables the computation of Granger causality (GC)^28^. GC has been shown to be an effective method of evaluating causal relationships between neuronal ensembles^27^. Thus, to verify the direction and strength identified by ir-PAC, we also calculated GC spectra of the same bipolar LFP recordings^37,38^ and averaged across all experiments under Dataset III (see Fig. 2), following the procedures described earlier^27^. GC revealed unidirectional information flow from DG to CA1 at theta frequency (~5 Hz in Fig. 7B) (GC = 0.94 ± 0.52), whereas GC in the opposite direction showed no significant causality (GC value = 0.02 ± 0.02). GC spectra and ir-PAC corresponded with each other in Fig. 7A,B. Furthermore, calculated from the same LFP recordings, the magnitude of ir-PAC between DG high-gamma amplitude and CA1 theta phase (DG → CA1) positively correlated with the magnitude of theta band DG → CA1 GC (Fig. 7C) with a correlation coefficient of 0.9012 (p = 0.0009). In contrast, as shown in Fig. 7D, there was no significant correlation (r = 0.4742, p = 0.1972) between the magnitude of CA1 → DG GC and ir-PAC between CA1 high gamma amplitude and DG theta phase (CA1 → DG). Here, the comparison between the new technique, ir-PAC, and the well-established analytical measure of directionality, GC, was enabled by the significant variability of DG → CA1 direction measurements in individual recordings within Dataset III (Fig. 8). ir-PAC was applied to unipolar wire recordings referenced to common reference in freely moving animals (Dataset II), whereas GC was applied to bipolar LFP recordings derived from 16-channel silicon probe across CA1 and DG in urethane-anesthetized rats (Dataset III). The first represents the main target of this application, the second represents the model allowing the most reliable GC estimations^27^. GC appeared more reliable at revealing significant DG → CA1 direction in all 9 recordings (Fig. 8B) whereas ir-PAC lead to significant estimate in 69% of (9 of 13, Fig. 8A) the recordings.

## Discussion

In this study we tested the idea that ir-PAC, namely, the high gamma amplitude of one LFP and the theta phase of another LFP, can be used to infer the direction and strength of rhythmic neural transmission. Specifically, we found significant ir-PAC between high-gamma amplitude of LFP signals recorded in networks known to generate theta oscillations and theta phase of LFPs recorded in downstream structures receiving input from these networks through well-established non-reciprocal anatomical projections. When theta and high-gamma components of the two LFP signals were switched, PAC showed no significant relationship, indicating that theta phase of the driver circuits and high-gamma amplitude of the receiver circuits are not coupled. These findings suggest that ir-PAC can be used to infer direction and magnitude of rhythmic drive between distinct brain networks.

After formulating the idea conceptually, we proceeded to test it in datasets containing recordings from distinct networks. The first dataset concerned the hippocampus-PFC network, a cortical network of two spatially segregated structures in which PFC neuronal firing and LFP were known to be modulated by hippocampal theta in a behavioral and task dependent manner^30,39–42^. Although theta rhythm can be transmitted to PFC and hippocampus from common sources or through a reciprocal connection through the thalamus, the dominant theta drive is carried by the non-reciprocal hippocampo-prefrontal pathway^43^. In the second dataset, concerning the DG and CA1 network within the hippocampus, theta is known to be generated in both recorded structures, by local mechanisms as well as common inputs, primarily from the medial septum and entorhinal cortex^31–33^. Communication between DG and CA1 is unidirectional, however, through the classic trisynaptic pathway, thus indicating that there should be significant ir-PAC between DG high-gamma amplitude and CA1 theta-phase, but not the other way around. This was also verified with GC, a well-established analytical measure of directionality, which revealed the same directionality, i.e., DG → CA1, and showed numerical correspondence between theta GC and ir-PAC values (Fig. 7C).

The interpretational framework for the ir-PAC results is based on the accumulating evidence that LFP recordings contain high-gamma components contributed by action potential transients in the underlying neural structure^17,20,21^. It should be acknowledged that postsynaptic potentials of true oscillations remain hard to separate from such spike “contamination”^20,44–46^. Most spectral analysis techniques can reveal multiple oscillations as spectral peaks riding on top of the characteristic background 1/f decay in power spectra, but are inadequate to handle transients best described as delta function or a Gaussian with small sigma contributing to LFP spectra in a wide frequency range. Using matching pursuit, however, a technique that allows decomposition of LFP into oscillatory (narrow-band) and transient (broadband) components, Ray and Maunsell (2011) demonstrated that such LFP transients were associated with spikes in specifically designed experiments in which gamma oscillations and spiking were anti-correlated in monkey visual cortex and could be controlled and thus be separated by changing the stimulation parameters. They also showed that although sharp transients most likely had power over the entire spectrum these could only be observed over ~50 Hz, due to masking at lower frequencies by the 1/f character of LFP spectra^20^. The 50–60 Hz cut off frequency was further supported by detailed analysis of firing rate and band power in different behaviors showing positive correlation in a wide gamma range of 50–180 Hz with peaks between 60–90 Hz (ref.^21^, their Fig. 2). PAC between theta and broadband higher gamma followed a similar pattern in the present study (i.e., significant PAC was broadly distributed above 55 Hz; Fig. 5). The 65–85 Hz frequency range chosen in our study for PAC calculation proved suitable to verify the hypothesis both for l-PAC within the same LFP recording and ir-PAC between LFPs in distinct structures, with the latter being exploited for directional information (Figs 3, 4).

Although PAC calculated from one signal does not give directional information, it is tacitly assumed in most studies (see however^47,48^ for examples showing the opposite) that low-frequency oscillations modulate high-frequency oscillations, not vice versa. In contrast, in the present application of PAC between two LFPs, we found that, it was the high-gamma amplitude of one signal which was controlling theta phase of the other. Assuming that high-gamma reflect population spiking, therefore the output activity of a neuronal ensemble, and that low frequency oscillations (e.g., theta) reflect somatic/dendritic processing, therefore the input activity of a neuronal ensemble, we conclude that ir-PAC, instead of testing the relationship between a pair of genuine network oscillations, connects the output of a driver and the input of a receiver network. Thus, periodic increase in spike activity at the theta frequency in the driver network will result in theta bursts in hippocampo-fugal pathways which elicit theta rhythmic postsynaptic membrane potential response from neurons in the target receiver network. This interpretation is further supported by the finding that ir-PAC can only be measured effectively when slow frequency oscillations (e.g., theta) in the driver are strong, i.e., over the threshold of generating enough action potentials on the output. Our results (Fig. 6) show that datasets with low theta power in DG and CA1, did not show significant coupling in any direction, across or within areas.

The interpretation of two-LFP PAC measurements supported by the present findings suggests that ir-PAC may serve as a suitable tool to infer directional information for low-frequency rhythmic drive between distinct networks. Similar to other widely used techniques analyzing inter-regional communication, e.g., coherence, its effectiveness however is strongly influenced by the exact anatomical wiring defining the recorded LFP^15^. In the laminar hippocampal datasets for example, which allow precise layer-specific localization of recordings (e.g., including the layer where CA3 theta input arrives in CA1^32,33,49–51^) and using bipolar constructs permitting the most reliable GC estimations^27^, parallel ir-PAC and GC analysis showed a highly significant positive correlation between the two variables (Fig. 7), suggesting that ir-PAC reveals not only the direction but also the strength of rhythmic neural transmission. In the widely used experimental preparation in which hippocampal LFP is monitored in less stringent conditions, i.e., using monopolar CA1 and DG wire recordings, ir-PAC still lead to significant estimate in ~70% of recordings and pointed in the correct direction in all remaining experiments (Fig. 8A), indicating that even when ir-PAC values failed to pass statistical threshold (thus deemed not statistically significant), the values may still be used to hint at the direction and strength of rhythmic neural transmission.

Theta-gamma PAC has become a widely used, powerful tool for understanding neural dynamics in both human and animal research. Gamma power in rat was found to be modulated by ongoing theta oscillations in rat hippocampus more than two decades ago^52^. More recently, PAC was tested in a range of cognitive tasks; for example, theta-gamma phase-phase coupling was found during maze exploration and REM sleep^53^, theta phase-gamma amplitude coupling strength increased during learning in rat hippocampus, thus contributing to memory processing^7^, nesting of gamma frequency oscillations within slow theta frequency in rat hippocampus indicated routing of information as a major function of gamma frequency in different bands^8^, high gamma power was found to be modulated by theta phase in human neocortex^12^, simultaneous maintenance of multiple items in working memory was observed to be accompanied by PAC in human hippocampus^14^, high frequency oscillation modulated by the type of low frequency oscillation is task dependent, as observed in human intracranial recording^13^. The interpretation proposed for local PAC, l-PAC, may shed further light on these studies in terms of low-frequency oscillatory activities in a neural network and the relation with the output activities from the network. Similarly, the directional information obtained from ir-PAC may add a further dimension to the interpretation of the few prior studies in which PAC was calculated across regions and showed results equivalent to this study, i.e., significant PAC between the amplitude of high-frequency hippocampal LFP and theta phase of extrahippocampal structures, but not vice versa, including PFC^1^ (their Fig. 6C) and striatum^54^ (their Fig. 5A).

Two further observations are in order. First, although ir-PAC only gives information of propagation of the low-frequency oscillation, it has important impact on coupling of genuine gamma oscillations in distinct networks. Gamma oscillations are difficult to synchronize over longer distances because of their low power (compared with delta, theta, alpha, beta, -cf. 1/f spectral background) and uncertainties and non-uniformity of conduction times which have progressively higher impact as the oscillation cycle is getting shorter and shorter^55^. Thus, gamma oscillations are considered local, i.e., generated by local networks in different cortical structures. Their synchrony is achieved by synchronizing low-frequency rhythms effortlessly carried over long distances entraining neuronal firing and establishing synchronized gamma oscillations on both ends of the pathway. Hippocampo-cortical communication is based for example on theta synchrony flexibly established between cooperating networks in a behavior and task-dependent manner serving precise cognitive functions^29^.

Second, while most PAC studies focus on nested gamma oscillations at lower frequencies (35–55 Hz)^7,56^, LFPs due to genuine oscillations do appear at high-gamma frequencies, as well. For example, Pernia-Andrade and Jonas^57^ demonstrated IPSPs in intracellular recordings of DG granule cells coherent with extracellular LFPs in the high-gamma range (76 ± 5 Hz). Cross-frequency coupling of real network oscillations were also shown in this range and even above 100 Hz, e.g. 50–90 and 90–150 Hz^53^, 100–140 Hz^8^, 80–150 Hz^12^, up to 140–180 Hz (related to genuine high frequency oscillations, or HFO^54,58,59^) indicating that these gamma oscillations may provide specific channels of communication separate from low-gamma oscillations^20,45^. Although unlikely, the contribution of high-gamma oscillations to the present results cannot be completely excluded. Coupling of gamma power and spike activity in the mixture of LFPs was shown in general^18,60–62^ and thus some of the bursting neuronal ensembles might be synchronized in the high-gamma range and co-vary with transients related to action potentials.

## Methods

All the surgical and other relevant aspects of the experimental procedure were approved by the Institutional Animal Care and Use Committee.

### Data acquisition

Experiments were performed on male Sprague–Dawley rats (Charles River Laboratories, MA) treated in accordance with NIH guidelines. All procedures were approved by the Institutional Animal Care and Use Committees of Beth Israel Deaconess Medical Center.

#### Dataset I

Rats were implanted with chronic EEG and EMG electrodes under ketamine-xylazine (70–80 and 10 mg/kg, respectively) anesthesia. Field potentials were recorded using surface screw electrodes placed at equidistant locations along a rostro-caudal axis, over the left frontal, parietal and occipital cortex (AP: 1.0, −2.5, −6.5 mm, Lat: 2.0, 2.5, 3.0 mm, respectively) and with fine wires in the prefrontal cortex (PFC) (AP: 3.2, Lat: 0.2, DV: 5.1 mm, on both sides) and hippocampus (AP: 3.7, Lat: 2.2, DV: 3.5 mm, on the right side). Daily recordings started 7–10 days after surgery. The rats were placed in a recording box in the morning, and cortical and hippocampal EEG, and neck muscle EMG were continuously recorded for 24 h without disturbing the animal^59,63^. Theta-intensive periods were selected from awake as well as REM sleep and recordings from the hippocampus and PFC were submitted to analysis (Fig. 2). A total of 6 recordings of awake and 6 recordings of REM from 6 animals (n = 12) were used for this dataset. Awake data and REM data were separately analyzed.

#### Dataset II

Rats were prepared for chronic recordings using surgical and recording techniques similar to Dataset I. Hippocampal LFPs were recorded in DG and CA1 regions of the hippocampus using a pair of twisted wires placed above and below the hippocampal fissure (AP: 3.7, Lat: 2.2, DV: 2.5 and 3.5 mm), verified by out-of-phase theta oscillations and later by histology^64–66^ LFPs from simultaneous CA1 and DG recordings during awake and REM sleep theta states were submitted to analysis (Fig. 2). A total of 13 different recordings (n = 13) from 4 animals were analyzed.

#### Dataset III

Hippocampal LFPs were recorded from rats under urethane anesthesia^67^ using a 16-channel linear multicontact electrode with 100 µm separation between contacts. The linear electrode was implanted in the dorso-ventral direction to cover a 1.5–2.5 mm segment across CA1, DG, and hilar regions^27,66^ (Fig. 2). CA1 and DG regions were identified by perforant path evoked potentials. Theta rhythm was elicited by high frequency (100 Hz) stimulation of the pontine reticular formation^27,66^. A total of 9 different recordings (n = 9) from 5 animals were analyzed.

### Data analysis

#### Preprocessing

A high pass filter (>1 Hz) and a notch filter centered at 60 Hz were applied to all datasets. For Granger causality (GC) analysis (Dataset III), LFP data was downsampled from 1 k Hz to 200 Hz.

#### Power spectral analysis

Power spectra were obtained using the Welch method of power spectral estimation where the window was 2 sec in duration with 50% overlap.

#### Phase amplitude coupling (PAC)

Each dataset consisted of continuous data segments of varying length. The unit of analysis for PAC were these segments of continuous recordings simply referred to as recordings or experiments. Animals may be in different behavioral states depending on the dataset. Dataset I was recorded from 6 animals during awake exploration and REM sleep. Dataset II was recorded from 4 animals during natural awake exploration and REM sleep. The data analyzed were recordings during which theta was present. Such recordings varied in length ranging from 50 sec to 1000 sec for Dataset I (n = 6 for awake exploration and n = 6 for REM) and for Dataset II (n = 13). Dataset III was recorded in 5 anesthetized rats where theta was elicited using brainstem stimulation. There were 9 stimulation experiments. In each experiment there were 10 to 20 stimulation episodes ranging from 5 sec to 10 sec in duration. The data from multiple stimulation segments in which theta occurred from each experiment was collectively referred to as a recording (n = 9). The PAC analysis method was the mean vector length - modulation index (MVL-MI) technique^12^. It has the following steps. First, consider two LFP signals. One was filtered in the high-gamma frequency range (65–85 Hz), denoted fA, and the other in the theta frequency range (4–8 Hz), denoted fp. From the Hilbert transforms of the filtered signals, the amplitude of high-gamma, denoted ${{\rm{A}}}_{{{\rm{f}}}_{{\rm{A}}}}$, and the phase of theta, denoted ${{\rm{\Phi }}}_{{\rm{fp}}}$, were extracted, and the complex-valued composite analytic signal ${\rm{Z}}({\rm{t}})={{\rm{A}}}_{{{\rm{f}}}_{{\rm{A}}}}\ast {{\rm{e}}}^{{{\rm{i}}{\rm{\Phi }}}_{{\rm{fp}}}}$ was formed. The modulation index (MI) was derived from the first moment (M_RAW_) of Z(t) where M_RAW_ was normalized before it was used as a metric of coupling strength. Second, 200 surrogate signals were created by offsetting the amplitude of gamma filtered signal ${{\rm{A}}}_{{{\rm{f}}}_{{\rm{A}}}}$ by a time lag ${\rm{\tau }}$. The data points in ${{\rm{A}}}_{{{\rm{f}}}_{{\rm{A}}}}$ were cyclically shifted after lag ${\rm{\tau }}$ randomly 200 times. Then the PAC measure was computed 200 times for 200 surrogate amplitude segments using the MVL-MI technique with the original theta phase filtered signal ${{\rm{\Phi }}}_{{\rm{fp}}}$ such that ${\rm{Z}}({\rm{t}},{\rm{\tau }})={{\rm{A}}}_{{{\rm{f}}}_{{\rm{A}}}}({\rm{t}}+{\rm{\tau }})\ast {{\rm{e}}}^{{{\rm{i}}{\rm{\Phi }}}_{{\rm{fp}}}({\rm{t}})}$. Third, normalized Z-score was obtained to yield M_NORM_ = (M_RAW_ − μ)/σ where μ was the mean of the surrogate lengths and σ was their standard deviation. M_NORM_ was used as a measure of interregional PAC or ir-PAC. For Local PAC or l-PAC, the filtering was done on the same LFP signal, and the other steps were the same. To compute PAC comodulogram, M_NORM_ was calculated between amplitude of LFP signal ranging from 1 to 100 Hz with 10 Hz bandwidth and 5 Hz step and phase of LFP signal ranging from 1 to 30 Hz with 2 Hz bandwidth and 0.5 Hz step.

#### Granger causality analysis

Parametric GC analysis was performed on Dataset III. Bipolar signals were derived from the theta generators in DG and CA1 respectively (Fig. 2) and the two bipolar signals were then subjected to AR modeling (see^27^) from which Granger causality spectral estimates were obtained. If the theta segment was 10 sec in duration it was further epoched into five non-overlapping 2 sec epochs which were treated as realizations of a stochastic process. If the theta segment was 5 sec in duration it was epoched into two non-overlapping 2.5 sec epochs which were treated as realizations of a stochastic process. The beginning of each epoch could be viewed as time 0. We note that in each experiment the stimulation episodes were of the same duration (5 sec or 10 sec depending on the animal). To eliminate the influence of common signals and volume conduction, bipolar derivation was taken, which was shown to be a critical step in the proper estimation of Granger causality between two neuronal ensembles^27^. For Datasets I and II, due to the electrodes applied, bipolar derivation was not possible to obtain. Thus, Granger causality analysis was not attempted on these two datasets.

#### Testing of statistical significance

To test whether the estimated Granger causality or the PAC (l-PAC or ir-PAC) modulation index was significantly greater than 0, we utilized a random permutation approach^68^. In this approach a baseline null-hypothesis distribution was constructed from which statistical significance threshold was derived. Each continuous data recording was divided into epochs of 2 second or 2.5 second in duration. For two time series within the recording the epoch index from one time series was permuted randomly against that from the other to create a synthetic dataset. Granger causality spectra and ir-PAC modulation index were derived from the synthetic dataset and the appropriate average value was taken. This random permutation procedure was repeated five hundred times to yield the null hypothesis distribution of GC and ir-PAC spectra for the respective statistical significance test. In both cases, values from the actual dataset were compared against the synthetic data distribution and considered significant only if they exceeded the 95th percentile value of the null hypothesis distribution (p < 0.05).

## Data Availability

The datasets generated during and/or analyzed during the current study are available from the corresponding author on reasonable request.
